# Acute Subdural Hematoma: A Rare Manifestation of Cerebral Venous Sinus Thrombosis

**DOI:** 10.7759/cureus.45519

**Published:** 2023-09-19

**Authors:** Satish Nirhale, Shalesh Rohatgi, Prajwal Rao, Pravin Naphade, Khushboo S Hatekar

**Affiliations:** 1 Neurology, Dr. D.Y. Patil Medical College and Research Center, Pune, IND

**Keywords:** cerebral venous sinus thrombosis (cvst), subdural hematoma (sdh), acute subdural hematoma, sdh with cvst, acute subdural hematoma with cvst

## Abstract

Cerebral venous sinus thrombosis (CVST) usually presents with headaches, seizures, and signs and symptoms of raised intracranial pressure (ICP). Risk factors for CVST commonly include hypercoagulable states such as pregnancy and the peripartum period, dehydration, vitamin B12 deficiency, hyper-homocysteinemia, coagulation factor deficiency, antiphospholipid antibody disease, oral contraceptive pill intake, etc. CVST with venous hemorrhagic infarction is commonly reported, but only a few cases have been reported in the literature of CVST presenting as SDH.

Here, we present a case of a 28-year-old female who presented with an acute onset of severe headache, vomiting, and bilateral papilledema on fundus examination. She had a prior history of oral contraceptive pill intake. An MRI brain venogram suggested CVST involving the superior sagittal sinus, right transverse, and a few cortical vein thromboses with subdural hematoma (SDH) in the frontal-parieto-temporo-occipital region on the right side. The patient was treated with anticoagulants and antiepileptics and had significant improvement in symptoms with the resolving SDH on subsequent scans.

## Introduction

Cerebral venous sinus thrombosis (CVST) is more commonly seen in a younger population, with a median age of 37 years [1]. Causes of cerebral venous thrombosis include several prothrombotic states (congenital or acquired), such as deficiencies in anti-coagulation-promoting proteins (factor V Leiden mutation, protein C and protein S resistance, prothrombin gene abnormalities, and antithrombin III deficiency), usage of oral contraceptives, pregnancy, malignancy, dehydration, trauma, inflammatory diseases, operative procedures, infections, and hematological conditions. The prognosis of CVST is better with the standard treatment line of management, which includes careful anticoagulation. Despite advances in the recognition of cerebral venous sinus thrombosis in recent years, the diagnosis of cerebral venous sinus thrombosis is still frequently overlooked or delayed as a result of diverse underlying risk factors, a wide spectrum of clinical symptoms, and its often sub-acute or lingering onset [2-3].

Here, we report a case of a 28-year-old female who was presented with signs and symptoms of raised intracranial pressure. She was diagnosed with CVST involving the superior sagittal sinus, right transverse, and few cortical vein thrombosis, with the rare manifestation of a subdural hematoma (SDH). The patient responded very well to anticoagulation treatment.

## Case presentation

A 28-year-old female with no previous comorbidities presented with a severe throbbing holocranial headache for 10 days. It was associated with two episodes of vomiting. She also had one episode of seizure, with a semiotic pattern of left upper limb focal tonic-clonic movement followed by generalization and a post-ictal phase lasting for 20 minutes. Additionally, she had a history of transient visual obscuration for the past seven days. She reported a history of oral contraceptive pill intake for a period of 10 days for an irregular menstrual cycle.

On examination, her vital parameters were within normal limits. Fundus examination revealed bilateral papilledema. She did not exhibit any other focal neurological deficits. Routine blood laboratory investigations, including serological markers and thyroid function tests, were normal. D-dimer and vitamin B12 levels were normal, and serum homocysteine was 12 mg/dl.

**Figure 1:**
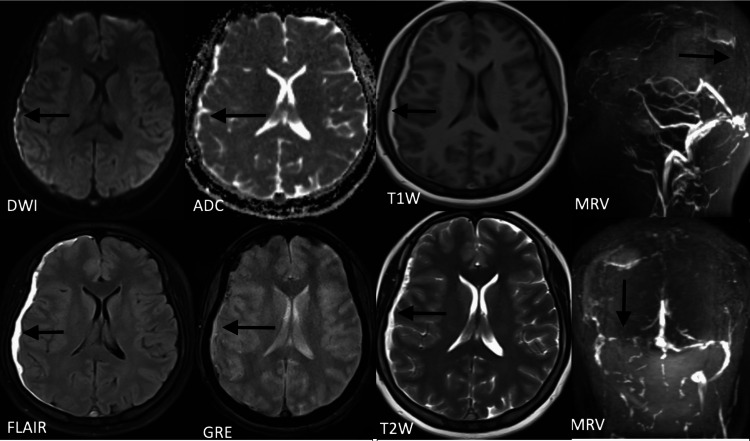
Magnetic resonance imaging of the brain (T2/FL) Air sequence showing crescent-shaped extra-axial hyperintensity and loss of flow void in superior sagittal sinus and few cortical vein thrombosis. Magnetic resonance imaging of the brain venogram suggests superior sagittal sinus, right transverse, and few cortical vein thrombosis. DWI: Diffusion-weighted imaging, ADC: Apparent diffusion coefficient, FLAIR: Fluid-attenuated inversion recovery, GRE: Gradient recalled echo, MRV: Magnetic resonance venogram.

MRI brain venogram revealed CVST involving the superior sagittal sinus, right transverse sinus, and a few cortical veins, along with a subacute subdural hematoma (SDH) in the frontal-parieto-temporo-occipital region on the right side (Figure 1). The patient was started on low molecular weight heparin (LMWH), along with antiepileptics, and was provided with hydration.

On further workup, the antinuclear antibody (ANA) test using the immunofluorescent method was weakly positive, but the ANA blot was negative. Additionally, the antiphospholipid antibody profile was negative. Protein C and S and genetic workup were advised.

**Figure 2:**
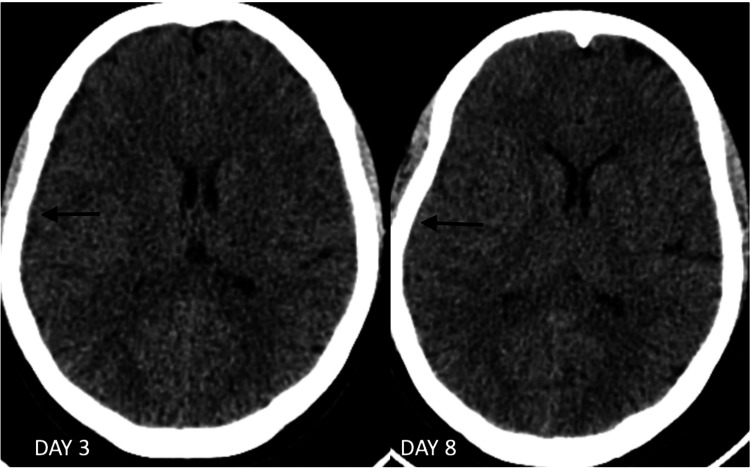
Computed tomography (CT) brain plains: on days three and eight of treatment.

On subsequent follow-up, a plain CT scan of the brain showed a resolving subdural hematoma along the right cerebral hemisphere (Figure 2). Subsequently, the patient was started on warfarin with a targeted international normalized ratio (INR) between 2 and 3, and the patient exhibited a dramatic improvement in symptoms.

## Discussion

The prevalence of stroke due to CVST is approximately 0.5%-1%, usually affecting a younger population [3]. The median age of those affected is 37 years, while our patient is 28 years old [1-3]. CVST is more prevalent in females compared to males, and our patient is also female [3]. The presentation of CVST commonly includes headaches, seizures, transient visual obscuration, and vomiting, all of which were observed in our patient [2-4]. Bilateral papilledema, a sign of raised intracranial pressure, is also reported in most patients, and it was present in our patient [3-4].

One of the risk factors in our case is a history of oral contraceptive pill intake, which is consistent with prior studies [3-5].The most affected sinuses in CVST are the superior sagittal sinus and transverse sinus, which align with our patient's condition [3-6].

However, there have been very few case reports of CVST presenting as subdural hematoma (SDH), which was the case with our patient [7]. The patient was treated with anticoagulation using LMWH bridging with warfarin, following standard treatment guidelines for CVST [8-10]. As a result, the patient showed a dramatic improvement in symptoms and resolution of the SDH. To support the hypothesis that CSVT directly causes SDH, further studies would need to identify cases of CSVT associated with SDH where there is no other plausible cause for the SDH [11]. 

## Conclusions

Acute SDH is a very rare complication of CVST, and only a few case reports have been documented in the literature so far. Currently, there are no clear guidelines for the management of SDH in the context of CVST. Anticoagulation remains the treatment of choice for CVST, but it should be administered with caution in such cases, accompanied by close neuromonitoring and neuroimaging. Further studies are necessary to establish appropriate management strategies for patients presenting with this combination of conditions.
